# 211. *Burkholderia Cenocepacia* Infections at Sites Other Than the Respiratory Tract, a Large Case Series from a Tertiary Referral Hospital in Lebanon

**DOI:** 10.1093/ofid/ofab466.413

**Published:** 2021-12-04

**Authors:** Housam Eddine Al Hariri, Rola Kwayess, Joya-Rita Hindy, Nada Youssef, Martine Elbejjani, Souha S Kanj

**Affiliations:** American University of Beirut Medical Center, Beirut, Beyrouth, Lebanon

## Abstract

**Background:**

*Burkholderia cenocepacia* has been described to cause mainly respiratory tract infections. We noticed an increased number of skin and soft tissue infections (SSTIs), bloodstream infections (BSIs) and osteomyelitis over the past years at our medical center.

**Methods:**

This is a retrospective chart review of 44 patients with documented *B. cenocepacia* infection at sites other than the respiratory tract diagnosed between 2005 and 2020 at the American University of Beirut Medical Center, a tertiary referral hospital for the Middle East region.

**Results:**

The nationalities of our patients were Iraqi (40.9%), Lebanese (34.1%), and Syrian (20.5%). Twenty six of the infections (59.1%) were hospital-acquired infections (HAIs). The most common infections were BSIs (17/44, 38.6%), then SSTIs as well as deep seated cysts and abscesses (16/44, 36.4%) and vertebral osteomyelitis (8/44, 18.2%). Half of the vertebral osteomyelitis were located in the lumbar, and 3 in the cervical region; 5 of these cases were native osteomyelitis. Sixteen patients (36.4%) had prior antibiotic intake within 30 days with ceftazidime, carbapenems and quinolones being the most common. All the patients received directed therapy for an average duration of 23.48 (+/- 37.779) days, and for 60 days for those with osteomyelitis. Combination regimens of 2 antibiotics (ceftazidime, quinolones, carbapenems, trimethoprim-sulfamethoxazole (TMP-SMX)) were used in 10 patients, whereas 24 received a single antibiotic. Thirty three patients (75%) were admitted to the hospital, 20 (45.5%) of which had an indwelling catheter and 12 (27.3%) were in the intensive care unit. Thirty two patients (96.9%) were discharged home. Susceptibility testing revealed 84.1%, 54.5%, 63.2%, and 65.9% susceptibility to ceftazidime, tetracycline, TMP-SMX, and carbapenems respectively.

Characteristics of patients

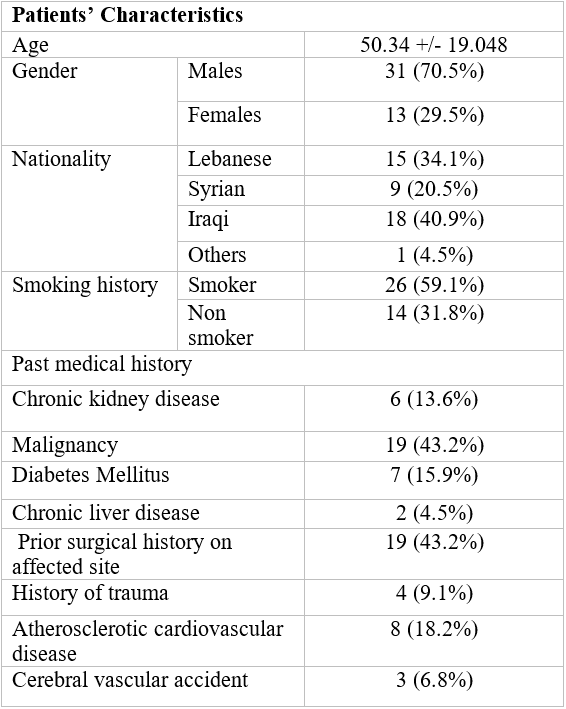

**Conclusion:**

*B. cenocepacia* BSIs, SSTIs, abscesses, and osteomyelitis were noted to be more common at our medical center as HAIs particularly in Iraqi and Syrian patients, raising the concern that countries at war might be at increased risk for such infections. Our susceptibilities results were consistent with the literature. Although *B. cenocepacia* is a resistant bacteria, the majority of our patients were successfully treated.

**Disclosures:**

**All Authors**: No reported disclosures

